# MCP-1-Induced Histamine Release from Mast Cells Is Associated with Development of Interstitial Cystitis/Bladder Pain Syndrome in Rat Models

**DOI:** 10.1155/2012/358184

**Published:** 2012-09-19

**Authors:** Jianwei Lv, Yiran Huang, Shiguo Zhu, Ganggang Yang, Yujian Zhang, Jing Leng, Juanjie Bo, Dongming Liu

**Affiliations:** ^1^Department of Urology, Renji Hospital, Shanghai Jiao Tong University School of Medicine, Shanghai 200127, China; ^2^Shanghai Institute of Medical Genetics, Jiaotong University School of Medicine, Shanghai 200040, China

## Abstract

*Objective*. Interstitial cystitis/bladder pain syndrome (IC/BPS) is characterized by overexpression of monocyte chemoattractant protein-1 (MCP-1) in bladder tissues and induction of mast cell (MC) degranulation. This study was undertaken to explore the mechanism of action of MCP-1 in the development of IC/BPS. *Methods*. A rat model of IC/BPS was developed by perfusing bladders of nine SPF- grade female Sprague-Dawley rats with protamine sulfate and lipopolysaccharide (PS+LPS). MCP-1 and histamine levels in bladder tissue and urine were detected by immunohistochemistry and ELISA. MC degranulation was measured by immunofluorescence techniques and chemokine (C-C motif) receptor 2 (CCR2) was assayed by flow cytometry. *Results*. Increased MCP-1 expression in bladder tissue and elevated MCP-1 and histamine levels were observed in the urine of LS+LPS-treated rats. This was accompanied by the expression of CCR2 on MC surfaces, suggesting MCP-1 may induce MC degranulation through CCR2. Exposure to LPS stimulated MCP-1 expression in bladder epithelial cells, and exposure to MCP-1 induced histamine release from MCs. *Conclusions*. MCP-1 upregulation in IC/BPS is one of possible contributing factors inducing histamine release from MCs. CCR2 is involved in the process of mast cell degranulation in bladder tissues. These changes may contribute to the development of symptoms of IC/BPS.

## 1. Introduction

Interstitial cystitis/bladder pain syndrome (IC/BPS) is a chronic inflammatory condition that is difficult to characterize without precise knowledge of the symptoms and histology that characterizes the disease [1]. Commonly observed symptoms include urinary frequency, urgency, and suprapublic pain that often drastically affect the quality of life of affected patients [2–4]. It is generally thought that the development and progression of IC/BPS is associated with infection, defects in bladder permeability, immune or neuroendocrine disorders, and genetic disorders related to visceral hypersensitivity [5–7]. Despite comprehensive characterization of symptoms and histology of IC/BPS in recent decades, its exact etiology remains unclear, limiting the development of effective therapeutic interventions.

Mast cells (MCs), long suspected to play a role in the onset of IC/BPS, are derived from specific precursors localized in the bone marrow [8]. These precursors are stimulated to maturation by local tissue microenvironmental factors that vary according to the tissue types [9]. MC stimulation has been shown to facilitate the degranulation and release of vasoactive, proinflammatory, and nociceptive mediators in the target tissue, most notably histamine, cytokines, and proteolytic enzymes [10]. These compounds increase the sensitivity of the sensory neurons, resulting in a positive feedback loop that further activates MCs and sustains the release of increasing concentrations of inflammatory cytokines [11]. This self-facilitating process is thought to be initiated by various factors, including extreme cold, drugs, neuropeptides, trauma, or toxins. These factors activate only small numbers of mast cells [12, 13] and the gradual onset of the disease process makes the causative factor difficult to identify. 

Full knowledge of the mechanisms which triggers IC/BPS has yet to be elucidated. However, previous studies have shown that a cascade of MC proliferation in bladder tissue is likely to be correlated with the onset of IC/BPS symptoms [14]. In support of this proposal, elevated levels of urinary histamine are commonly observed in patients with IC/BPS [15] and are often used as a diagnostic factor for the condition [1]. Other studies have demonstrated that histamine released by MCs plays a key role in neural sensitization that is responsible for the bladder and urinary pain associated with IC/BPS [16]. Histamine levels have therefore been used as a biomarker for IC/BPS in genetic studies [17]. 

Determination of the mechanism underlying the regulation of histamine release in MCs is required to further clarify the role of MCs in IC/BPS and provide potential targets for future development of therapeutic agents designed to reduce the impact of the condition on quality of life. 

Monocyte chemoattractant protein-1 (MCP-1) is a chemokine that plays diverse and important roles in many different inflammatory conditions, including rheumatoid arthritis, atherosclerosis, and neuroinflammatory disease [18, 19]. It has been reported that patients with IC/BPS exhibit elevated MCP-1 in both the urine and bladder tissues, suggesting a possible association between MCP-1 and the severity of IC/BPS [20]. MCP-1 has also been implicated in the mediation of MC degranulation [21, 22], suggesting that it plays a critical role in MC activation processes, including those that occur over the course of IC/BPS. While the role of MCP-1 has been widely explored in other inflammatory diseases, further research is required to fully clarify the relationship between upregulation of MCP-1 and the release of histamine in amounts significant enough to trigger symptomatic IC/BPS. 

Based on the previously suggested hypothesis that MCP-1 plays a critical role in the induction of histamine release from MCs in bladder tissues of patients diagnosed with IC/BPS, the current study was designed to quantify the expression of MCP-1 in bladder tissues and the levels of both MCP-1 and histamine present in urine using rat models of IC/BPS. Previous studies suggest that IC/BPS is likely to produce significant increases in MCP-1 expression and corresponding increases in the levels of MCP-1 and histamine observed in the urine. Furthermore, this study aims to provide evidence of degranulation in MCs potentially associated with the expression of surface chemokine (C-C motif) receptor 2 (CCR2). Additionally, *in vitro *studies were constructed to assess the effect of LPS treatment on MCP-1 release in bladder epithelial cells, likely agents in the acceleration of histamine release from MCs. Further evidence for the mechanistic relationship between MCP-1 and the release of histamine in MCs associated with IC/BPS will provide a critical groundwork necessary for the further development of interventions for the diagnosis, prevention, and treatment of IC/BPS.

## 2. Materials and Methods

### 2.1. Animal Models and Sampling

Nine female Sprague-Dawley rats of SPF grade (250–300 g) were purchased from the Laboratory Animal Department of the Jiaotong University Medical School, Shanghai, China. All animal studies were conducted in accordance with Shanghai Jiaotong University guidelines for ethical animal study.

The rats were divided into a Model group (*n* = 6), representing IC/BPS subjects, and a Control group (*n* = 3), representing normal subjects. The IC/BPS rat model was established by bladder perfusion with protamine sulfate and lipopolysaccharide (PS+LPS) (Sigma, USA) according to previously described methods [23]. Briefly, the rats were anesthetized by intraperitoneal injection with sodium pentobarbital (40 mg/kg), followed by immediate disinfection of the external urethral orifice with a 70% ethanol alcohol plus iodine tincture. The bladder of each rat was subsequently emptied with a PE-50 catheter (AHMSIC, USA), and resultant urine samples were stored in liquid nitrogen for analysis. One milliliter of protamine sulfate (10 mg/mL in PBS) (Sigma, USA) was injected into the bladder. After 45 min the bladder was drained and flushed three times with PBS. This was followed by injection of 1 mL of LPS (750 *μ*g/mL) (Sigma, USA) into the bladder, which was maintained for 30 min. At this time point the control group was injected an equal volume of PBS. 

The vesica urinaria were harvested from each animal after 5 days. Bladder tissues were stored in liquid nitrogen, prior to being weighed and ground. Protein lysate (1 mL lysate per 20 mg tissue) and protease inhibitor were subsequently added to the tissue. The bladder tissue was ground for another 1 min and placed on ice for 1 min. The resultant tissue was then ground three times and centrifuged at (800 rpm/min). 

### 2.2. Cell Culture

Human urothelial cells (SV-HUC-1) and rat MCs (RBL-2H3) were purchased from the Cell Bank of the Type Culture Collection of the Chinese Academy of Sciences (Shanghai, China). SV-HUC-1 cells were cultured in F12 K medium supplemented with 10% fetal bovine serum (FBS) (Sigma, USA), 2 mM L-glutamine, 1.5 g/L sodium bicarbonate, and lipopolysaccharide (LPS; Sigma, USA) at concentrations of 0, 10, 100, and 500 ng/mL. Expression of MCP-1 was evaluated at 24, 48, and 72 h.

RBL-2H3 cells were cultured in 85% Earle's balanced salt solution (Hyclone, USA) supplemented with 1.5 g/L sodium bicarbonate, 0.1 mM nonessential aminoacids, 1.0 mM sodium pyruvate, 2 mM L-glutamine, 15% FBS (Sigma, USA), and human MCP-1 (Shanghai Winhong Biology Technology Co., Ltd., China) at concentrations of 0, 10, and 100 ng/mL. The histamine content of cells was detected at 24, 48, and 72 h.

### 2.3. ELISA Detection of MCP-1 and Histamine

MCP-1 concentrations and histamine levels in samples isolated from bladder tissues, urine, and the supernatant of cultured cells were detected using the rat MCP-1 ELISA kit (Thermo, USA) and the histamine ELISA kit (Shanghai Westang Biology Technology Co., Ltd., China), respectively, in accordance with the instructions provided by the manufacturer.

### 2.4. Determination of CCR2

RBL-2H3 cells were rinsed twice with PBS containing 0.1% BSA and 0.05% sodium azide. The samples were then adjusted to a density of 1 × 10^7^ cell/mL in PBS solution. 100 *μ*L of cell suspension was incubated with APC-labeled mouse anti-rat CCR2 antibody (BD Pharm Mingen, USA) at 4°C under dark conditions for 30 min. The cells were then rinsed twice with PBS and resuspended in 200 *μ*L PBS prior to flow cytometric analysis. The expression of CCR2 was determined using a FACS Calibur flow cytometer (BD Biosciences, NJ) with isotype-matched negative controls. 

### 2.5. Immunohistochemical Detection of MCP-1 Expression in Bladder Tissues

Bladder tissues were excised, fixed in 4% formaldehyde for 24 h, embedded in paraffin, and sectioned. MCs were stained with toluidine blue (Beijing Bo Orson Biological Technology Co., Ltd., China) in order to identify degranulation. MCP-1 expression was assessed using immunohistochemistry. Briefly, bladder tissue sections were deparaffinized, rehydrated, and fixed with a solution of methanol-0.3% H_2_O_2_ for 30 min at room temperature. The antigen was prepared by rapid cooling for 3 min after highly compressed heating. The primary antibody, rabbit anti-rat polyclonal antibody to MCP-1 (Abcam, UK), was added and incubated overnight at room temperature. At the end of this process, bladder tissue sections were incubated with biotinylated antibody (anti-rat IgG) (Sigma, USA) and horseradish streptavidin (Beijing Chi Biological Technology Co., Ltd., China) for 1 h at room temperature. Finally, the samples were incubated with diaminobenzidine (DAB) (Dr Wuhan's Biological Engineering Co., Ltd., China) for coloration and counter stained with hematoxylin (Beijing Chi Biological Technology Co., Ltd., China) for 2 min, producing a distinctive blue tint in the nuclear region and a brown color in positively stained cells. 

### 2.6. Detecting MCP-1 Expression in Mast Cells with Immunofluorescence

MCP-1 expression in MCs was assayed using immunofluorescence. Briefly, tissue slices were rinsed three times with PBS and incubated with anti-MCP-1 polyclonal antibody, rabbit polyclonal antibody to rat MCP1 (Abcam, UK), at 4°C overnight. MCs were stained with tryptase [24, 25] (H-9) (1 : 100) (Thermo, USA). After rinsing with PBS, tissue slices were incubated with Alexa Fluor 488 goat anti-HRP IgG (H+L) (Sigma, USA) or HRP anti-mouse with Alexa Fluor 555 (1 : 500) (Sigma, USA) at 37°C for 2 h. Tissue slices were subsequently mounted in a Vectashield with DAPI (Beijing Chi Biological Technology Co., Ltd., China) and viewed with a Zeiss LSM710 Confocal fluorescence microscope (Carl Zeiss GmbH, Germany). 

### 2.7. Statistical Analysis

The data are presented as means ± standard deviations (mean ± SD). Between-group differences were analyzed using the Mann-Whitney *U* test. *P*-values less than 0.05 were considered statistically significant (*P* < 0.05). All data were analyzed using the SPSS version 18.0 statistical software (IBM, USA).

## 3. Results

### 3.1. MCP-1 Expression in Bladder Tissues and Urine

**Figure 1:**
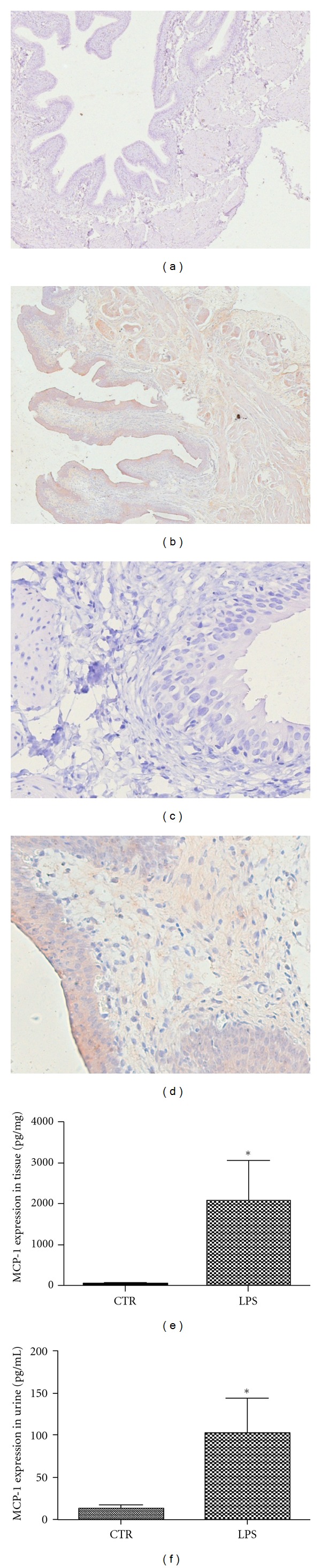
MCP-1 immunodetection. LPS-treated rats showed increased expression of MCP-1 in bladder tissues. (a) and (c) Control group (*n* = 3); (b) and (d) Model group (*n* = 6). ((a) and (b): ×40; (c) and (d): ×400). ELISA results indicate that MCP-1 levels in bladder tissue (e) and urine (f) from the Model group were elevated compared to levels observed in the Control group (*P* < 0.05).

 First we compared the expression of MCP-1 in bladder tissue and urine between Control and Model groups. The immunohistochemical results indicated that MCP-1 protein expression in bladder tissues was significantly elevated in the Model group (Figures 1(b) and 1(d)) compared with the Control group (*P* < 0.05). The MCP-1 concentration in bladder tissue was 2054 ± 991.8 pg /mg in the Model group compared with 35.67 ± 27.23 pg/mg in the Control group (Figure 1(e)). As shown in Figure 1(f), MCP-1 levels in urine were also significantly higher in the Model group (101.7 ± 42.27 pg/mL) than in the Control group (12.7 ± 4.24 pg/mL; *P* < 0.05).

### 3.2. Histamine Release in Bladder Tissues and Urine

**Figure 2:**
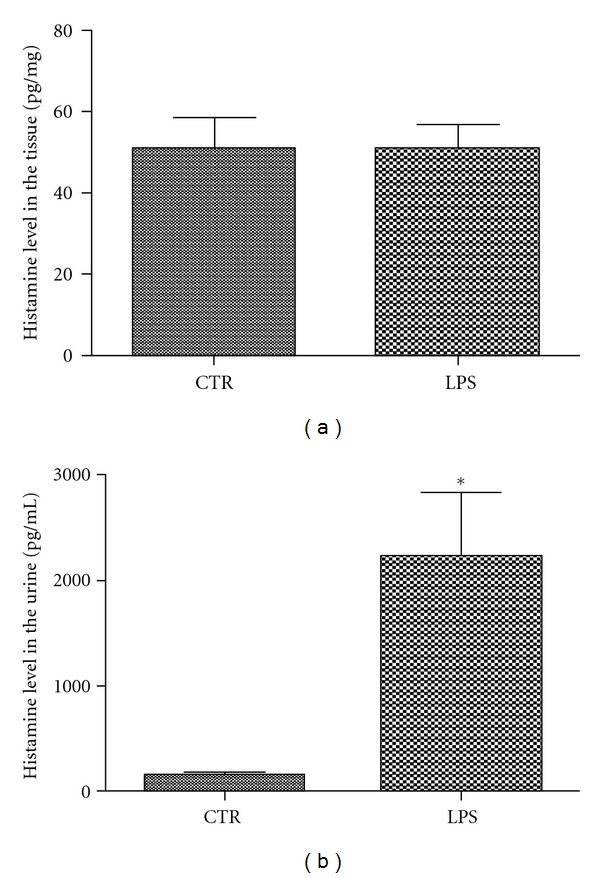
ELISA results for histamine analysis. (a) Histamine levels in bladder tissues of the Model group (*n* = 6) show no significant difference with those of the Control group (*n* = 3) (*P* = 0.796). (b) Histamine levels were significantly elevated in urine from the Model group compared with the Control group (*P* < 0.05).

To test whether the upregulation of MCP-1 in bladder tissue by PS+LPS induced histamine release, we measured the levels of histamine both in bladder tissue and urine. As shown in Figure 2(a), the histamine release in bladder tissues was comparable in the Model (50.93 ± 5.88 pg/mg) and Control groups (51.01 ± 7.63 pg/mg; *P* = 0.796). However, significantly higher levels of histamine were observed in urine samples from the Model group (2225 ± 618.3 pg/mL) than from the Control group (147.1 ± 42.33 pg/mL; *P* < 0.05) (Figure 2(b)).

### 3.3. Degranulation of MCs and MCP-1 Expression in Bladder Tissues

**Figure 3:**
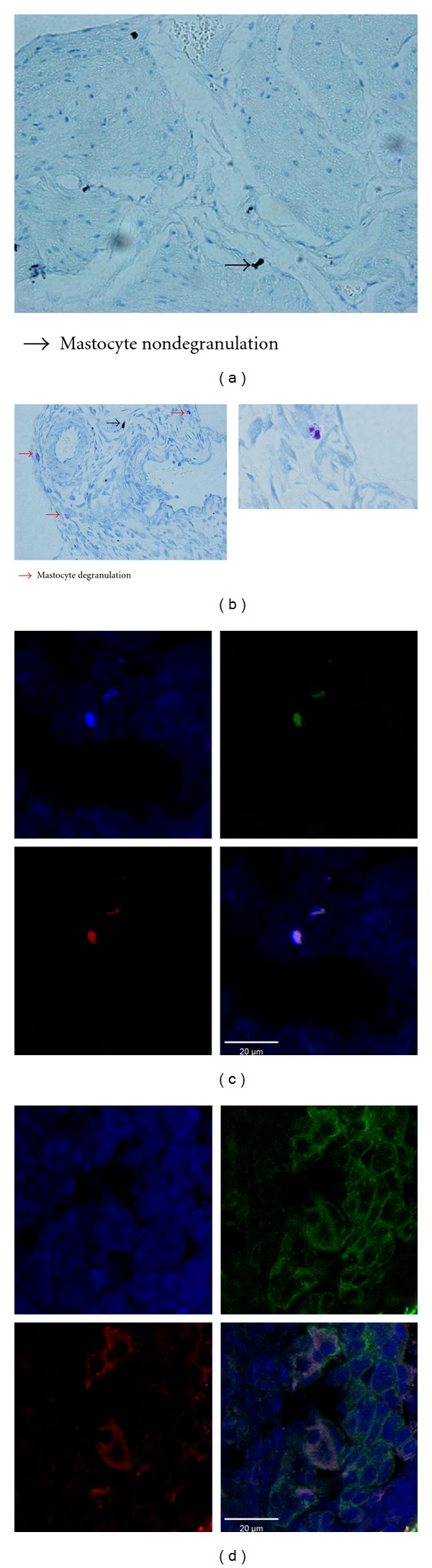
LPS-induced degranulation of mast cells. (a) Control group (*n* = 3); (b) Model group (*n* = 6). The mast cells with or without degranulation are indicated with red or blue arrows, respectively. Double-labeled immunofluorescence microscopy showing MCP-1 interactions with mast cells in bladder tissues. (c) Control group (*n* = 3); (d) Model group (*n* = 6). Nuclei are colored blue by DAPI; green fluorescence indicates MCP-1 protein immunostaining; red fluorescence indicates MC immunostaining. Scale bars in the immunofluorescence images represent 20 *μ*m.

To verify whether PS+LPS-induced IC/BPS was associated with the release of histamine from mast cells in bladder tissue, we investigated mast cell degranulation using immunohistochemistry. MCs in the bladder tissue exhibited obvious signs of degranulation in the Model group (Figure 3(b)) but no indication of degranulation was observed in the Control group (Figure 3(a)). These findings suggest that the increased level of histamine in the urine of the Model group may be linked with increased degranulation of MCs in bladder tissue. Immunochemistry also showed that MCP-1 protein was localized in mast cells, suggesting a strong relationship between MCP-1 and degranulation of MCs (Figure 3(d)).

### 3.4. CCR2 Expression in Bladder Tissues and MC Surfaces

**Figure 4:**
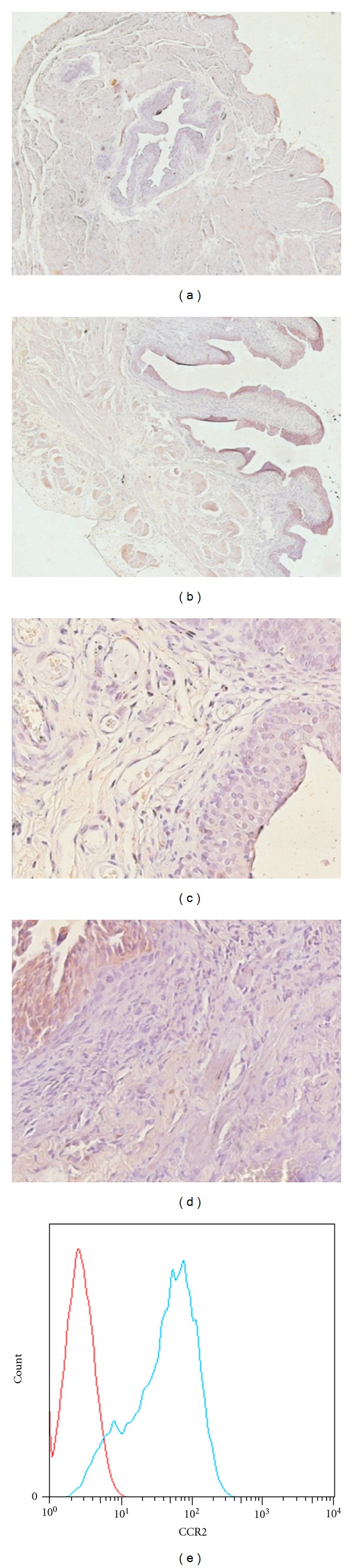
LPS-induced CCR2 receptor expression in bladder tissues. (a) and (c) Control group; (b) and (d) LPS group. CCR2 receptors were detected with immunohistochemistry ((a) and (b) ×40; (c) and (d) ×400). (e) LPS-increased CCR2 receptor expression on the surface of mast cells detected by flow cytometry.

Given that increased MCP-1 expression in bladder tissue and histamine release from mast cells were observed in PS+LPS-induced IC/BPS rats, we hypothesized that MCP-1 may induce the degranulation of mast cells within bladder tissue through CCR2. Immunohistochemistry showed increased expression of CCR2 in bladder tissues from the Model group compared with the Control group (Figures 4(a)–4(d)). CCR2 receptor expression on MC surfaces was further verified using flow cytometry (Figure 4(e)).

### 3.5. LPS-Stimulated MCP-1 Release in Bladder Epithelial Cells

**Figure 5:**
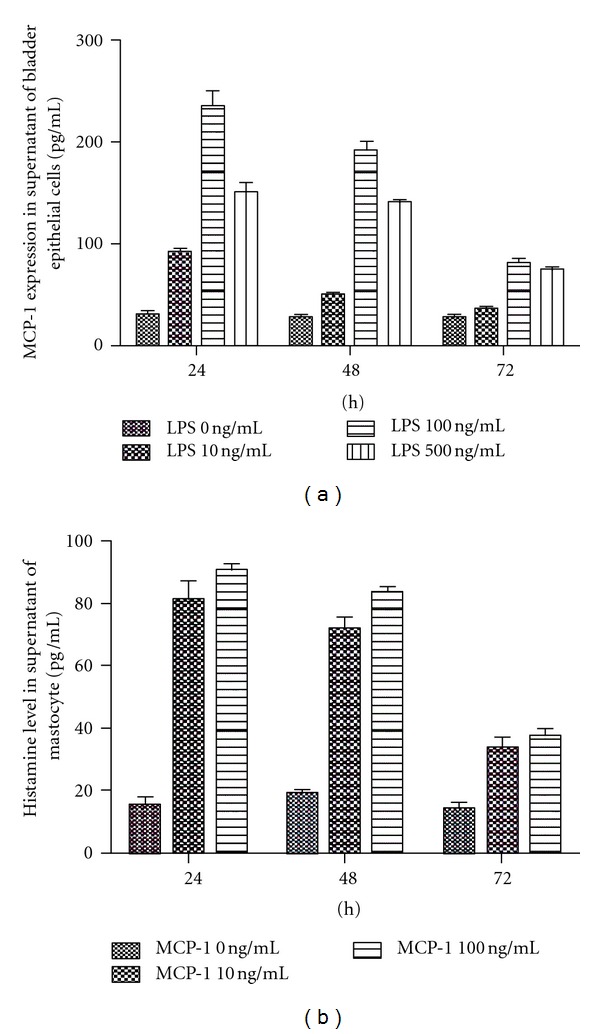
LPS-stimulated MCP-1 release from bladder epithelial cells in a dose- and time-dependent manner (a). MCP-1 induced histamine release from MCs (b). MCP-1 and histamine in the supernatant of bladder epithelial cells and mast cells were detected with ELISA (*n* = 3).

 Although we have observed that MCP-1 expression was increased in bladder tissue in LPS-induced IC/BPS rats, it is uncertain that if LPS can induce MCP-1 production directly *in vitro*. *In vitro* bladder epithelial cells exposed to LPS at concentrations of 10, 100, and 500 ng/mL for 24, 48, and 72 h demonstrated a clear time- and dose-dependent increase in MCP-1 levels in the supernatant. The maximum increase of MCP-1 was achieved after exposure to LPS 100 ng/mL for 24 h. This increase was maintained for 48 h, but was no longer detectable 72 h after exposure (Figure 5(a)).

### 3.6. MCP-1-Stimulated Histamine Release from MCs

Increased expression of MCP-1 in bladder tissue was accompanied by elevation of histamine release in LPS-induced IC/BPS rats, suggesting that MCP-1 may directly stimulate histamine release from mast cells. To test this hypothesis, mast cells were cultured in the presence or absence of MCP-1 at concentrations of 10 or 100ng/mL and histamine contents of the supernatant were measured after 24, 48, and 72 h.The maximum stimulation of histamine release was seen after exposure to 10 ng/mL MCP-1 for 24 h (Figure 5(b)). No significant differences in histamine release were observed between 10 and 100 ng/mL after 24 or 48 h of exposure (Figure 5(b)), suggesting that MCP-1 may stimulate the histamine release from mast cells directly. 

## 4. Discussion

IC/BPS is associated with a number of symptomatic sequelae that are poorly understood on a mechanistic level. The current study provides evidence suggesting that MCP-1-induced increases in histamine release are associated with CCR2 surface proteins on MCs and are involved in the development and progression of IC/BPS. 

It has been shown that PS can induce rat bladder epithelial injury, resulting in altered urothelial permeability. Subsequent injection of LPS is thought to penetrate the interstitium and initiate the release of proinflammatory cytokines, leading to the formation of experimental cystitis in rats [23]. The rat model using PS+LPS applied in the current study is symptomatically consistent with IC/BPS symptoms in humans, including the presence of bladder inflammation, edema formation, and leukocyte infiltration [23]. The findings of the current study provide further evidence for the likelihood of the involvement of MCs in human IC/BPS, as well as a potential mechanism for this action.

In the IC/BPS rat model, an increase in MCP-1 expression was observed in both the bladder tissue and urine, a phenomenon accompanied by increased CCR2 expression in bladder tissues and on MC surfaces. We also observed, increased MC degranulation and histamine release in the rats with experimentally induced IC/BPS, but, surprisingly, the histamine levels in bladder tissues were found to be similar to those in the control group. 

The levels of urinary histamine were significantly higher in rats with IC/IBC than in control rats, demonstrating that increased expression of MCP-1 and elevated histamine release from MCs may play a critical role in the development of IC/BPS. *In vitro* exposure to LPS resulted in elevation of MCP-1 in bladder epithelial cells. It has previously been demonstrated that moderate increases in the expression of MCP-1 mRNA in bladder tissues of patients with IC/BPS were directly correlated with the severity of clinical symptoms [26], providing evidence for the role of MCP-1 in the development of the disease process. In support of these previously published findings we were able to demonstrate that incubation of mast cells with MCP-1 was accompanied by increased histamine release. 

Additional studies have confirmed that MCP-1 is a potent chemokine that causes mast cell recruitment and activation *in vitro* [26], providing a possible link between MCP-1 and inflammatory conditions such as IC/BPS. It has also been reported that mast cells promote cystitis pain and induce pathophysiological changes in the bladder tissue of mice with neurogenic IC/BPS mice induced by pseudorabies virus (PV). The mechanism underlying these changes has been linked to increased histamine production associated with tumor necrosis factor-alpha (TNF-*α*) [27], which is a similar process to that observed in the current study. 

Increased release of MCP-1 has been observed in human detrusor smooth muscle cells stimulated with IL-4 [17] suggesting that locally produced chemokines, cytokines, and growth factors are able to recruit and destabilize MCs in smooth muscle tissue, such as that of the bladder. Because of their demonstrable impact on smooth muscle tissues, compounds that stimulate chemokines, cytokines, and growth factor production are likely to play a significant role in the pathophysiology of IC/BPS. These observations all support the possible role of MCP-1 in IC/BPS demonstrated by the current study. Increases in histamine release in urine, coupled with increasing MC activation in the bladder tissue of patients with IC/BPS highlights the significance of histamine promotion in the development and progression of symptoms. Though no clear evidence previously demonstrated the relationship between MCP-1 and histamine release from MCs in IC/BPS, the current results provide a strong indication that MCP-1 can, in fact, play a key role in inducing increasing levels of histamine release in MCs. Furthermore, evidence has provided that the mechanism likely involves CCR2 proteins on the MC surface, providing a potential future target for development of therapeutic treatments for the condition. 

While further studies will be required to determine all of the varied mechanistic factors associated with the development and progression of IC/BPM, the current study demonstrates that MCP-1 expression in tissues of the bladder is associated with concurrent increases in histamine release from MCs. The induction of MCP-1 expression in bladder tissues may thus play a critical role in the stressors associated with IC/BPS development, wherein significant degranulation and histamine release from MCs are initiated and propagated. Furthermore, the current study provides an initial basis for for MCP-1 stimulation of degranulation and histamine release in MCs, providing potential future targets for clinically applicable treatment and prevention of IC/BPS.
